# Genome-wide identification of small heat-shock protein (*HSP20*) gene family in grape and expression profile during berry development

**DOI:** 10.1186/s12870-019-2031-4

**Published:** 2019-10-17

**Authors:** Xiao-Ru Ji, Yi-He Yu, Pei-Yi Ni, Guo-Hai Zhang, Da-Long Guo

**Affiliations:** 1 College of Forestry, Henan University of Science and Technology, Luoyang, 471023 Henan Province China; 2 Henan Engineering Technology Research Center of Quality Regulation and Controlling of Horticultural Plants, Luoyang, 471023 Henan Province China

**Keywords:** Grape, HSP20, Gene family, Genome-wide analysis, H_2_O_2_

## Abstract

**Background:**

Studies have shown that *HSP20* (heat-shock protein 20) genes play important roles in regulating plant growth, development, and stress response. However, the grape *HSP20* gene family has not been well studied.

**Results:**

A total of 48 *VvHSP20* genes were identified from the grape genome, which were divided into 11 subfamilies (CI, CII, CIII, CV, CVI, CVII, MI, MII, ER, CP and PX/Po) based on a phylogenetic analysis and subcellular localization. Further structural analysis showed that most of the *VvHSP20* genes (93.8%) had no intron or only one intron, while genes that clustered together based on a phylogenetic tree had similar motifs and evolutionarily conserved structures. The HSP20s share a conservedα-crystalline domain (ACD) and the different components of the ACD domain suggest the functional diversity of VvHSP20s. In addition, the 48 *VvHSP20* genes were distributed on 12 grape chromosomes and the majority of *VvHSP20* genes were located at the proximal or distal ends of chromosomes. Chromosome mapping indicated that four groups of *VvHSP20* genes were identified as tandem duplication genes. Phytohormone responsive, abiotic and biotic stress-responsive, and plant development-related cis-elements were identified from the *cis*-regulatory elements analysis of VvHSP20s. The expression profiles of *VvHSP20s* genes (*VvHSP20–1*, *11*, *14*, *17*, *18*, *19*, *20*, *24*, *25*, *28*, *31*, *39*, *42,* and *43*) were largely similar between RNA-Seq and qRT-PCR analysis after hydrogen peroxide (H_2_O_2_) treatment. The results showed that most *VvHSP20s* were down-regulated by H_2_O_2_ treatment during fruit development. *VvHSP20s* genes were indeed found to be involved in the grape berry development and differences in their transcriptional levels may be the result of functional differentiation during evolution.

**Conclusions:**

Our results provide valuable information on the evolutionary relationship of genes in the *VvHSP20* family, which is useful for future studies on the functional characteristics of *VvHSP20* genes in grape.

## Background

As one of the most important cultivated fruit crops in the world, grape has high economic value. ‘Kyoho’ is a tetraploid interspecific hybrid and mid-late ripening grape cultivar derived from a cross between *Vitis vinifera* x *Vitis labrusca,* which is widely cultivated in China. Our previous studies on ‘Kyoho’ have shown that hydrogen peroxide (H_2_O_2_) treatment could promote the early ripening of ‘Kyoho’ grape, causing it to ripen 20 days earlier than the control [1, 2]. Other studies in tomato [3] and pear [4] have also demonstrated that H_2_O_2_ is associated with fruit development. H_2_O_2_ is an early component of the thermal signal pathway, which is a necessary condition for the activation of heat-shock protein 20 (HSP20) synthesis [5]. In addition, the response of *HSP20s* to H_2_O_2_ has also been revealed in tomato and rice, where H_2_O_2_ was shown to induce the expression of mitochondrial *HSP22* and chloroplast *HSP26*, respectively [6, 7]. It has been reported that HSP21 could protect photosystem II (PSII) from oxidative stress, promote color change during fruit ripening, and play a key role in the transformation of chloroplasts to pigment mother cells during fruit ripening [8].

The expression of HSPs is activated or increased under high temperature stress. According to molecular weight and sequence homology, HSPs can be divided into five families, which include HSP100, HSP90, HSP70, HSP60, and HSP20 [9, 10]. Among them, the molecular weights of HSP20 proteins are between 15 and 42 kDa, and are thus considered small HSPs. In some plant tissues, HSP20s comprise the largest proportion of HSPs [9]. HSP20s possess a typical conserved domain, known as the α-crystalline domain (ACD), which contains a conserved 80–100 amino acid sequence, a compact β-strand structure, and two conserved regions (CRs): CR I with β2, β3, β4, and β5; and CR II with β7, β8, β9, and a β6 loop [11]. HSP20s can prevent the damage of proteins caused by environmental stress and help them to fold or degrade [12, 13]. Thus, HSP20s are the important parts of cellular molecular chaperones.

In plants, *HSP20* genes are involved in many developmental processes and responses to abiotic stresses [14, 15]. Under heat stress, HSP20s can prevent the aggregation and irreversible denaturation of heat-denatured proteins, which ensures that other proteins can perform normal functions at high temperature, providing a strong basis for improving the heat resistance of plant organs. HSP20s have been shown to be located in mitochondria, cytoplasm, and endoplasmic reticulum [16].

The number of *HSP20* genes in plants is about four times greater than that in animals [17]. For example, 19, 35, 39, 42, 44, 51 members of the *HSP20* gene family were respectively investigated in Arabidopsis (*Arabidopsis thaliana*) [11], pepper (*Capsicum annuum* L.) [18], rice (*Oryza sativa*) [19], tomato (*Solanum lycopersicum*) [20], watermelon (*Citrullus lanatus* L.) [21], and soybean (*Glycine max*) [22]. To date, *HSP20* gene family members in grape have not been identified. Therefore, this study aims to elucidate the composition, gene structure, evolution, and expression of the grape *HSP20* gene family, in an attempt to characterize structural and functional features, and to establish a foundation for further utilization of plant HSPs.

## Results

### Genome-wide identification of *VvHSP20* gene family in grape

A total of 61 *VvHSP20* genes were obtained by Hidden Markov Model (HMM) analysis. The presence of an ACD domain was confirmed by submitting the protein sequences to CDD, Pfam, and SMART database. The sequences without the typical ACD domain were discarded. A total of 48 sequences were retained and confirmed as grape HSP20 after removing the sequences with a molecular weight beyond the 15–42 kDa. Detailed information on physicochemical properties of these HSP20s are listed in Table 1. The length of the VvHSP20 proteins varied from 136 (VvHSP20–47 and VvHSP20–48) to 365 amino acids (VvHSP20–41); the molecular weights of VvHSP20s were from 15.27 kDa (VvHSP20–30) to 40.59 kDa (VvHSP20–41). The predicted pI values of VvHSP20s ranged from 4.68 (VvHSP20–41) to 9.48 (VvHSP20–20).

**Table 1:** Features of VvHSP20 genes identified in grape

Gene name	Sequence ID	ORF Length (bp)	Chr	Chromosome Position	Length (aa)	MW (KDA)	pI	ProtComp
*HSP20–1*	VIT_01s0010g02290.t01	549	1	19,257,410–19,258,339	182	20.74	5.42	Chloroplast
*HSP20–2*	VIT_02s0154g00480.t01	606	2	5,248,542–5,250,041	201	22.45	9.24	Mitochondrial
*HSP20–3*	VIT_02s0154g00490.t01	606	2	5,255,141–5,256,154	201	22.55	9.11	Mitochondrial
*HSP20–4*	VIT_04s0008g01490.t01	471	4	1,219,942–1,220,465	156	17.34	5.94	Cytoplasmic
*HSP20–5*	VIT_04s0008g01500.t01	459	4	1,221,915–1,222,579	1521	16.69	6.84	Cytoplasmic
*HSP20–6*	VIT_04s0008g01510.t01	471	4	1,223,278–1,224,822	156	17.40	5.77	Cytoplasmic
*HSP20–7*	VIT_04s0008g01520.t01	471	4	1,224,823–1,225,354	156	17.58	5.58	Cytoplasmic
*HSP20–8*	VIT_04s0008g01550.t01	471	4	1,237,576–1,240,002	156	17.41	5.94	Cytoplasmic
*HSP20–9*	VIT_04s0008g01570.t01	501	4	1,244,737–1,246,530	166	18.60	5.95	Cytoplasmic
*HSP20–10*	VIT_04s0008g01580.t01	471	4	1,248,569–1,249,249	156	17.42	6.62	Cytoplasmic
*HSP20–11*	VIT_04s0008g01590.t01	468	4	1,251,984–1,252,699	155	17.29	5.94	Cytoplasmic
*HSP20–12*	VIT_04s0008g01610.t01	477	4	1,255,490–1,256,222	158	18.14	6.33	Cytoplasmic
*HSP20–13*	VIT_04s0008g01620.t01	480	4	1,257,262–1,257,741	159	18.42	8.46	Cytoplasmic
*HSP20–14*	VIT_06s0004g05770.t01	435	6	6,524,201–6,524,870	144	16.31	6.93	Cytoplasmic
*HSP20–15*	VIT_06s0009g01090.t01	948	6	12,540,124–12,542,550	315	34.98	8.58	–
*HSP20–16*	VIT_08s0032g00100.t01	579	8	3,034,519–3,035,221	192	21.52	8.45	–
*HSP20–17*	VIT_08s0058g00210.t01	447	8	8,901,763–8,902,216	148	16.88	5.81	Nuclear
*HSP20–18*	VIT_09s0002g00640.t01	483	9	440,684–441,359	160	17.89	6.3	Cytoplasmic
*HSP20–19*	VIT_09s0002g06790.t01	702	9	6,710,140–6,711,133	233	26.31	7.78	Mitochondrial
*HSP20–20*	VIT_12s0028g01390.t01	780	12	2,044,541–2,046,498	259	28.71	9.48	–
*HSP20–21*	VIT_12s0035g01910.t01	753	12	22,228,715–22,229,897	250	28.39	7.94	Endoplasmic reticulum
*HSP20–22*	VIT_13s0019g00860.t01	429	13	2,689,279–2,690,178	142	15.81	6.75	Peroxisomal
*HSP20–23*	VIT_13s0019g02740.t01	456	13	3,999,388–4,000,084	151	17.17	5.81	Nuclear
*HSP20–24*	VIT_13s0019g02760.t01	423	13	4,003,325–4,003,954	140	15.8	6.77	Cytoplasmic
*HSP20–25*	VIT_13s0019g02770.t01	456	13	4,006,363–4,007,091	151	17.1	5.81	Nuclear
*HSP20–26*	VIT_13s0019g02780.t01	456	13	4,015,394–4,016,080	151	17.02	5.8	Nuclear
*HSP20–27*	VIT_13s0019g02820.t01	456	13	4,036,190–4,036,907	151	17.12	5.81	Nuclear
*HSP20–28*	VIT_13s0019g02840.t01	456	13	4,043,383–4,044,010	151	17.09	5.54	Nuclear
*HSP20–29*	VIT_13s0019g02850.t01	456	13	4,048,636–4,049,360	151	17.05	5.8	Nuclear
*HSP20–30*	VIT_13s0019g02920.t01	411	13	4,108,657–4,109,160	136	15.27	5.7	Cytoplasmic
*HSP20–31*	VIT_13s0019g02930.t01	483	13	4,112,675–4,113,430	160	18.17	6.78	Cytoplasmic
*HSP20–32*	VIT_13s0019g03000.t01	483	13	4,149,244–4,149,995	160	18.15	7.93	Cytoplasmic
*HSP20–33*	VIT_13s0019g03010.t01	435	13	4,151,427–4,155,706	144	16.37	9.21	Cytoplasmic
*HSP20–34*	VIT_13s0019g03050.t01	498	13	4,180,057–4,183,444	165	19.23	6.46	Cytoplasmic
*HSP20–35*	VIT_13s0019g03090.t01	483	13	4,195,524–4,196,187	160	18.17	5.43	Cytoplasmic
*HSP20–36*	VIT_13s0019g03160.t01	483	13	4,227,250–4,227,937	160	18.02	7.94	Cytoplasmic
*HSP20–37*	VIT_13s0019g03170.t01	480	13	4,234,111–4,234,852	159	18.19	6.17	Nuclear
*HSP20–38*	VIT_14s0128g00280.t01	750	14	2,945,472–2,949,352	249	26.84	5.82	–
*HSP20–39*	VIT_16s0022g00510.t01	627	16	11,604,847–11,606,213	208	23.74	5.61	Mitochondrial
*HSP20–40*	VIT_16s0098g01060.t01	684	16	21,339,160–21,340,093	227	25.03	6.35	Chloroplast
*HSP20–41*	VIT_18s0072g00490.t01	1098	18	19,691,814–19,692,987	365	40.59	4.68	–
*HSP20–42*	VIT_18s0089g01270.t01	561	18	29,188,982–29,189,738	186	21.13	5.89	Cytoplasmic
*HSP20–43*	VIT_19s0014g05050.t01	579	19	5,376,784–5,377,821	192	22.39	5.35	Cytoplasmic
*HSP20–44*	VIT_19s0085g01050.t01	441	19	23,631,036–23,631,743	146	16.44	5.9	Cytoplasmic
*HSP20–45*	VIT_18s0001g01570.t01	492	18_random	2,135,300–2,136,089	163	18.28	6.33	Nuclear
*HSP20–46*	VIT_18s0001g01610.t01	480	18_random	2,183,110–2,184,273	159	18	5.74	Nuclear
*HSP20–47*	VIT_00s0707g00010.t01	411	unknow	34,510,489–34,511,388	136	15.69	4.89	Nuclear
*HSP20–48*	VIT_00s0992g00020.t01	411	unknow	37,397,842–37,398,790	136	15.7	5.01	Nuclear

### Phylogenetic analysis of *VvHSP20* genes

**Fig. 1:**
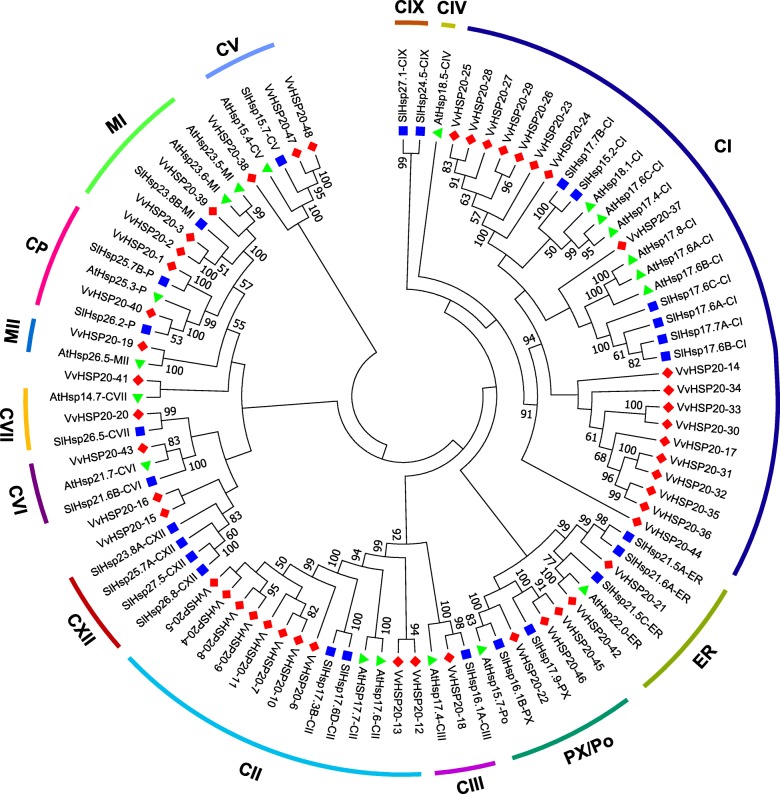
Phylogenetic tree of HSP20 proteins from grape and other plants. Phylogenetic tree of HSP20 proteins from grape and other plants including *Arabidopsis thaliana* and *Solanum lycopersicum* was constructed using MEGA7.0 based on the NJ method; bootstrap was 10,000 replicates. Percentage bootstrap scores of > 50% were displayed

An unrooted Neighbor-Joining (NJ) phylogenetic tree was constructed based on the alignment of amino acid sequences of HSP20 from grape, Arabidopsis, tomato (Fig. 1). In total, 19 sequences from Arabidopsis, 26 sequences from tomato, and 48 sequences from grape were assessed in the phylogenetic tree. According to the phylogenetic and the subcellular localization analysis, the grape HSP20 protein are divided into 11 subfamilies (CI, CII, CIII, CV, CVI, CVII, MI, MII, ER, CP, and PX/Po) (Fig. 1, Table 1). Clustering of the subfamilies in grape is largely consistent with the subcellular localization, i.e., the proteins in the same cluster were located in the same subcellular sites. Specifically, six HSP20 subfamilies (CI-CVI), MTI and MTII subfamilies, CP, ER and PX /Po localize to the cytoplasm/nucleus, mitochondria, chloroplast, endoplasmic reticulum and peroxisome, respectively. The 93 HSP20s were classified into 14 distinct subfamilies, except for the unclassified VvHSP20s (VvHSP20–15, VvHSP20–16, VvHSP20–38, and VvHSP20–41), the subcellular localization of which could not be predicted using the online tool Protcomp. Most of the VvHSP20s, including 33 out of 44, were classified into CI–CVII, which suggested that the cytosol may be the primary functional site of plant HSP20s.

### Characterization of the amino acid sequences and gene structure of VvHSP20s

**Fig. 2:**
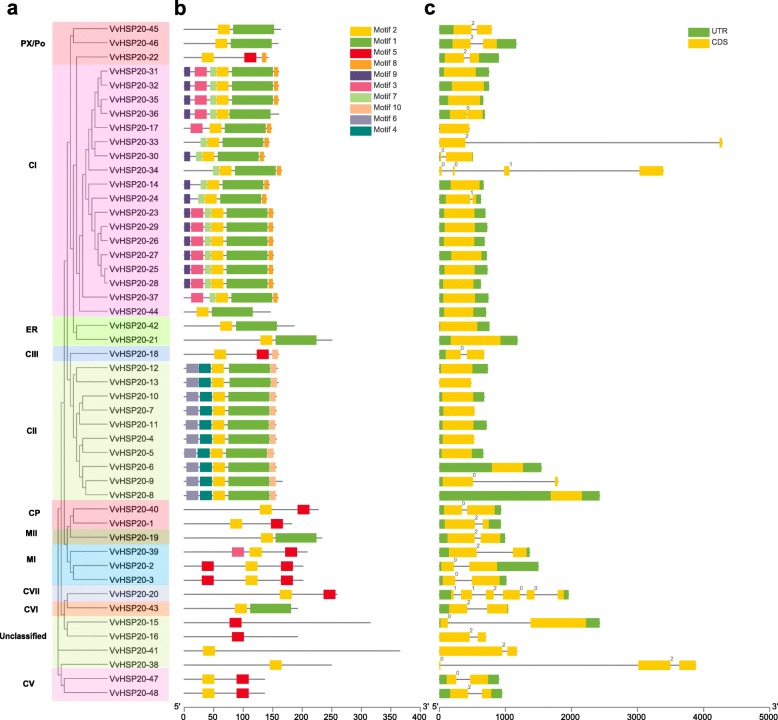
Phylogenetic tree, gene structure and domain analyses of VvHSP20s. **a** Phylogenetic tree of VvHSP20s was constructed with clustalx software. **b** Domain analyses of VvHSP20 proteins. Different color boxes represented the different types of motifs. **c** Gene structure of *VvHSP20s*. CDS sequences are represented by yellow round-corner rectangles and introns by grey lines, UTRs are shown with green boxes

**Fig. 3:**
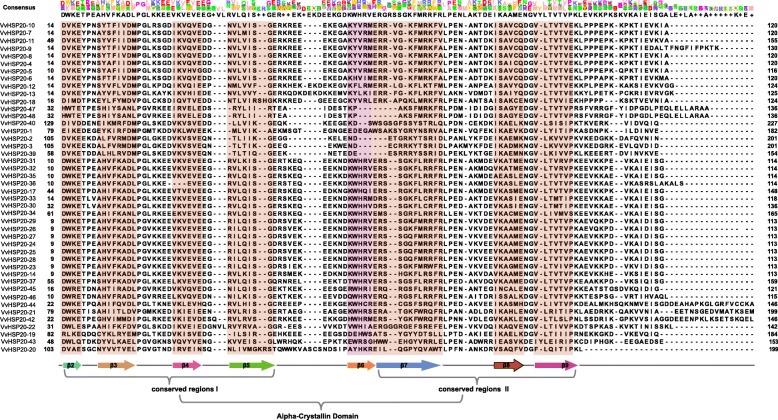
The alignment of ACDs of HSP20s in grape. Names of all members are shown on the left side of the figure. Each predicted β-plated sheet is shown for shadow. The primary structure of the ACD, including the conserved regions I (CRI), II (CRII), and β 6-loop, is shown at the bottom of the figure

As shown in Fig. 2a, 48 VvHSP20s were divided into 11 subgroups, except for the unclassified HSP20 (VvHSP20–15, VvHSP20–16, VvHSP20–38 and VvHSP20–41). Ten conserved motifs of VvHSP20 proteins were identified by the MEME website and listed in Table 2. The lengths of these conserved motifs ranged from 6 to 60 amino acids (Fig. 2b, Table 2). ACD consists of two conserved regions, CRI of β2, β3 and β4, and CRI of β7, β8 and β9, separated by a variable length hydrophilic region β6 loop (Fig. 3). VvHSP20–2, 3, 39, 40, 47 and 48 lacked the β6-loop. VvHSP20–36 lacked the β-strands 4. The different components of the ACD domain suggest functional diversity among VvHSP20s. The same group of VvHSP20 proteins in the phylogenetic tree had the same motif, which indicated that they were highly conserved.

**Table 2:** Motif sequences identified by MEME tools

Motif	Length (aa)	Sequence
1	70	VEEGRILQISGDRSVEKEEKNDKWHRVERSSGKFMRRFRLPENVKVDEVKAAMENGVLTVTVPKAEVQKP
2	21	DWKETPEAHVFKADLPGLKKE
3	21	NNMFDLWDPFQDFPFTGGALS
4	21	KSVSAPTRTYVRDAKAMAATP
5	21	MIDIDGISAGYEDGVLTVTVP
6	21	MMGFDSPLFSALQHMLDATDD
7	11	GETSAFANTRI
8	8	VKAIDISG
9	11	MSLIPSFFGGR
10	11	KKPKTIEVKIA

Next, we analyzed gene structure in order to better understand *HSP20s.* Among the *VvHSP20s* genes, 24 (50.0%) were intronless, and 21 genes (43.8%) possessed one intron. *VvHSP20–38* (2 introns), *VvHSP20–34* (3 introns), and *VvHSP20–20* (5 introns) had two or more introns (Fig. 2c). Genes of the same subgroup had the same intron phase, which indicated that the structure was quite conserved over evolution.

### Chromosomal location and gene duplication of *VvHSP20*

**Fig. 4:**
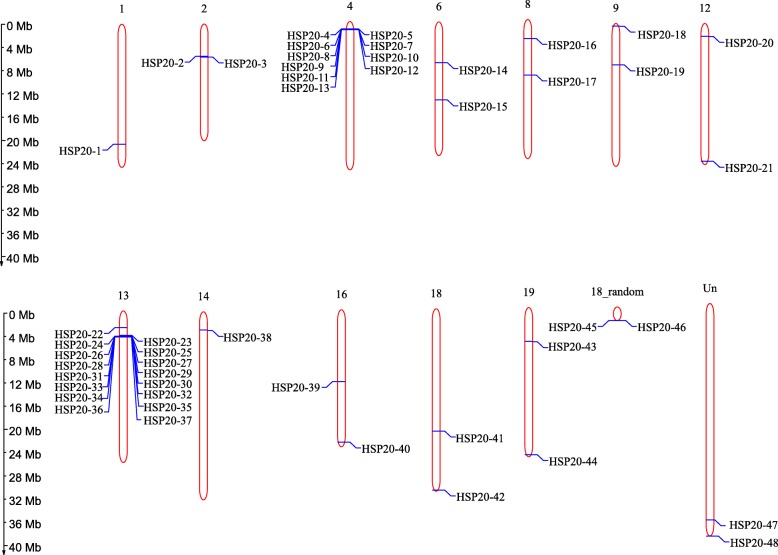
Chromosomal locations of *VvHSP20* genes on grape chromosomes. Blue lines indicated gene position

The 48 *VvHSP20* genes were distributed on 12 grape chromosomes (Fig. 4). Most of the *VvHSP20* genes were present on chromosome 4 (10 genes) and chromosome 13 (16 genes), while each of the remaining 10 chromosomes had one or two genes. Both tandem and segmental duplication contribute to the production of gene families during the process of evolution. Thus, potential duplication events of *VvHSP20* genes were analyzed. In total, four groups of *VvHSP20* genes (*VvHSP20–2*, *3*; *VvHSP20–4*, *5*, *6*, *7*, *8*, *9*, *10*, *11*; *VvHSP20–23, 24*, *25*, *26*, *27*, *28*, *29*, *30*, *31*, *32*, *33*, *35*, *36* and *VvHSP20–47*, *48*) were identified as tandem duplication genes (Additional file 1: Figure S1). Furthermore, none of the genes were suggested to be products of segmental duplication. Based on the above results, we inferred that tandem duplication played an important role in the expansion of the *VvHSP20* family in grape.

### Analysis of cis-element in *VvHSP20* gene promoters

**Fig. 5:**
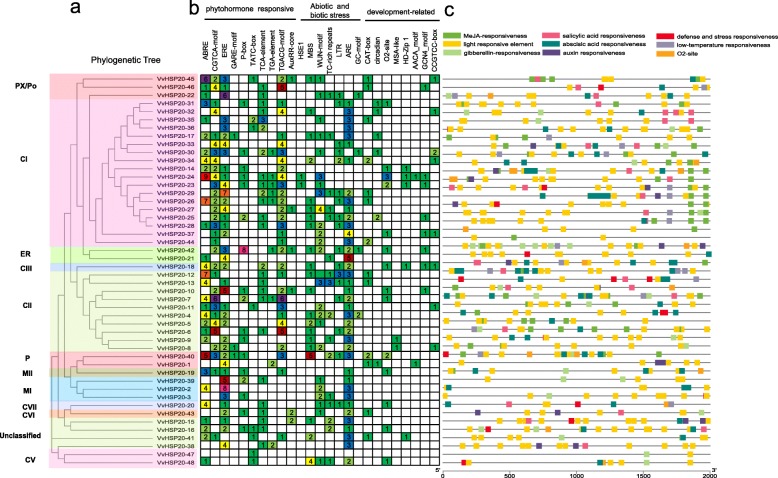
Investigation of cis-acting element numbers in *VvHSP20* genes. **a** The phylogenetic tree of VvHSP20s was constructed using Muscle module and neighbor-joining (NJ) method with 1000 bootstrap replicates implemented in MEGA7. Displaying of different background colors to distinguish the clades and subclades. **b** The different colors and numbers of the grid indicated the numbers of different promoter elements in these *VvHSP20* genes. **c** The different colored block represented the different types of cis-acting elements and their locations in each *VvHSP20* gene

To understand the possible role of cis-regulatory elements of *VvHSP20*, the promoter sequences (comprising − 2000 bp upstream of the translation start site) of 48 *VvHSP20* genes were submitted into PlantCARE to detect the cis-elements. Three categories of cis-elements, including phytohormone responsive, abiotic and biotic stress-responsive, and plant development-related cis-elements were identified and are shown in Fig. 5. Among the three categories of cis-elements, the phytohormone responsive category accounts for the highest proportion. In this category, cis-acting elements were widely present in the promoter region, including auxin responsive (TGA-element and AuxRR-core), gibberellin-responsive elements (GARE-motif, P-box, and TATC-box), ethylene-responsive (ERE), MeJA-responsive (TGACG-motif and CGTCA-motif), abscisic acid-responsive (ABRE), and salicylic acid-responsive (TCA-element). Among these elements, ABRE and ERE accounted for the largest part of the phytohormone responsive category. In the abiotic and biotic stress-responsive category, stress response-related cis-elements, such as HSE1 (heat stress), WUN motif (wound-responsive element), TC-rich repeats (stress response), LTR (low temperature-responsive), ARE (anaerobic induction), and GC-motif (anoxia) were detected. In the last category, plant development-related elements, including meristem expression (CCGTCC-box and CAT-box), circadian, zein metabolism regulation (O2-site), cell cycle regulation (MSA-like), differentiation of the palisade mesophyll cells (HD-Zip 1), and endosperm expression (AACA_motif and GCN4_motif) were identified. In addition, most of the *VvHSP20* genes possessed W boxes and MYB binding sites, including CCAAT-boxes.

### Expression patterns of *VvHSP20s* in response to H_2_O_2_ treatment

**Fig. 6:**
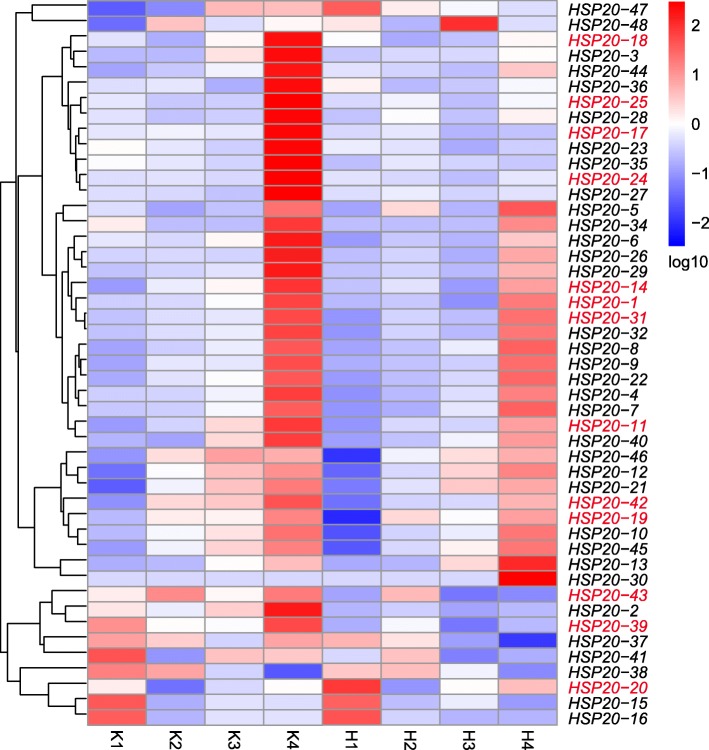
Relative transcriptional expression levels of *VvHSP20* from RNA-seq data in the H_2_O_2_ treatment and the control. Data were plotted after the Z-score normalization across the row based on absolute FPKM values of each gene at different development stages. The colors vary from blue to red representing the scale of the relative expression levels. ID in Red indicate genes selected for qRT-PCR analysis

There is a close relationship between gene expression and function. To determine the functions of *VvHSP20s* in grape, a heatmap of 47 *VvHSP20* genes was constructed using FPKM values from RNA-Seq data in control and H_2_O_2_-treated berries of ‘Kyoho’ (Fig. 6, sampling period is described in Materials and Methods and Table 3). The expression level of *HSP20–33* was extremely low and not detected by RNA-Seq analysis during fruit development. Most *VvHSP20s* were down-regulated after treatment, especially at the fourth period. However, the opposite trend was also observed for a few genes, including *HSP20–13*, *HSP20–20*, and *HSP20–30*. These results indicated that most of the *VvHSP20* genes responded to H_2_O_2_ treatment, and the response mechanisms of different *VvHSP20* genes to H_2_O_2_ were different.

**Table 3:** The sampling date of grape berries in this study

Development stage (dpa)	Sampling time	Code	
		control	Treated
35	6.12	K1	H1
45	6.21	K2	H2
55	7.1	K3	H3
65	7.12	K4	H4

dpa: Days post anthesis

**Fig. 7:**
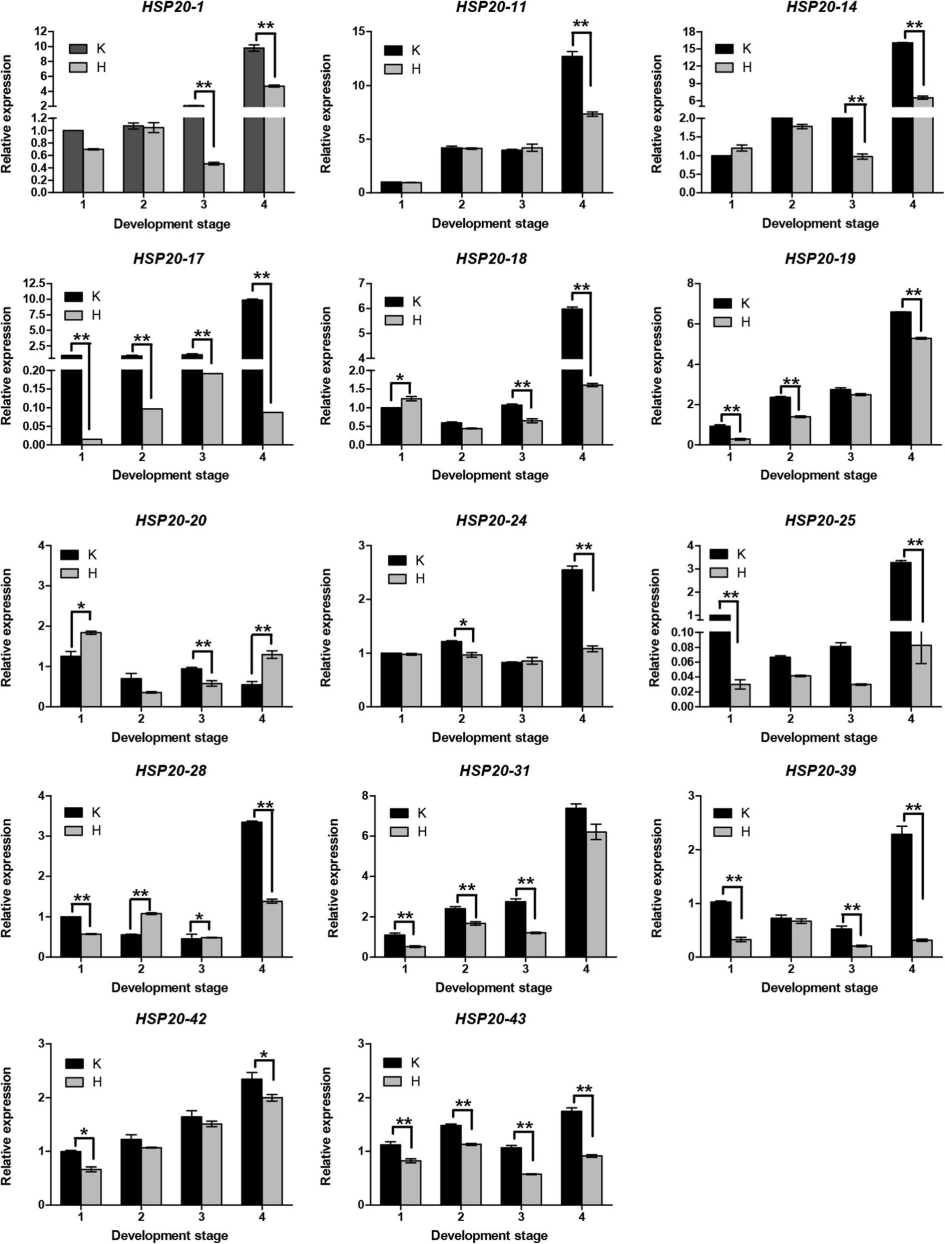
Expression profiles of *VvHSP20s* from qRT-PCR in the H_2_O_2_ treatment and the control. The x-axis represented different sampling date, while relative expression levels for the y-axis. Data represented the mean of three biological replicates. Error bars represented standard deviations from three independent technical replicates. And the expression level of K1 was used as the calibrator. The asterisks indicate the significant level (*P < 0.05, **P < 0.01) based on a Duncan’s multiple range test

Based on the statistical significance of the gene expression levels from the RNA-Seq analysis and the partitioning of the clusters of genes from the phylogenetic analysis, 14 differentially expressed *VvHSP20* genes were selected to be further validated by qRT-PCR in response to control and H_2_O_2_ treatment (Fig. 7). Consistent with the RNA-Seq data, the expression level of most genes decreased after the treatment. Besides *HSP20–31*, the relative expression levels of the remaining 13 genes were extremely down-regulated at the fourth period. It is worth noting that *VvHSP20–17* and *VvHSP20–25* were hardly expressed after treatment. Similar expression patterns were revealed within the tandem duplicated gene groups (*VvHSP20–25* and *VvHSP20–28*). The similar expression patterns indicated that the tandem duplicated *VvHSP20* genes had similar functions and structures. Members of the CI subgroup (*VvHSP20–24*, *VvHSP20–25*, *VvHSP20–28*, and *VvHSP20–31*) had similar expression patterns after the treatment, which suggested that they had similar functions in response to H_2_O_2_ treatment.

### Expression patterns of ABA-related genes in response to H_2_O_2_ treatment

**Fig. 8:**
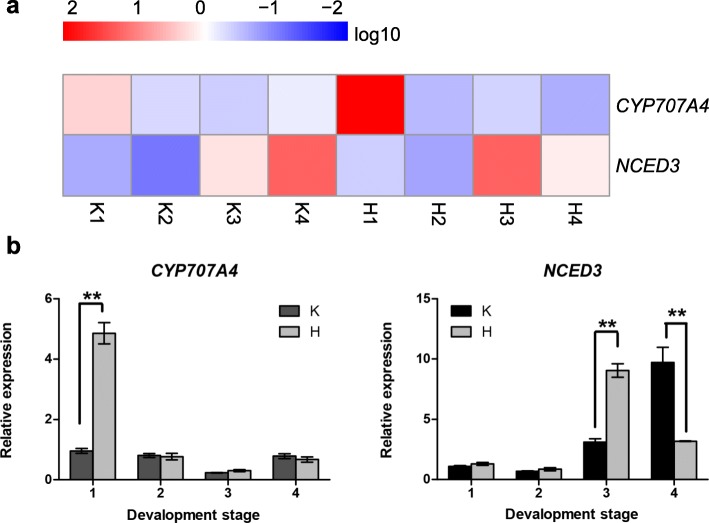
Expression profiles of ABA-related genes in the H_2_O_2_ treatment and the control. **a** The expression pattern of ABA-related genes from RNA-seq data. **b** Expression patterns of ABA-related genes from qRT-PCR. The x axis represented different sampling dates, while the y axis indicated relative expression levels. The data represent the average of three biological replicates. The error bar represents the standard deviation of three independent techniques. The expression quantity of K1 was used as calibrator. The asterisk indicates the significance level based on the Duncan multiple range test (*P < 0.05, **P < 0.01)

It is well known that ABA plays an important role in grape [23, 24]. In the previous study [1], H_2_O_2_ treatment was shown to promote the early fruit ripening of ‘Kyoho’. To further explore the role of ABA in this process, RNA-Seq and qRT-PCR were performed to examine the expression analysis of ABA-related genes. As shown in Fig. 8, the expression patterns of the ABA synthesis-related gene (*NCED3*) and degradation-related gene (*CYP707A4*) were different following H_2_O_2_ treatment. Compared with the control, the expression level of *NCED3* reached the highest level at veraison (H3 stage), then decreased at the H4 stage. On the contrary, the expression level of the *CYP707A4* gene increased rapidly after treatment and reached its lowest level at veraison. The changes in the expression levels of ABA-related genes indicated that H_2_O_2_ may regulate fruit development possibly through control of ABA catabolism and biosynthesis.

## Discussion

Fruit ripening is known to be regulated by a balance between reactive oxygen species (ROS) formation and detoxification by antioxidant enzymes [25, 26]. ROS causes senescence by accumulation of superoxide anion (O_2_^.-^) and hydrogen peroxide (H_2_O_2_) during fruit ripening [3]. H_2_O_2_ not only acts as a stress inducing factor, but also as a signaling molecule. Imbalance between ROS generation and removal can lead to oxidative stress in aerobic organisms [27, 28]. Previous studies on H_2_O_2_ signaling have identified a number of genes that are regulated by H_2_O_2_ levels [29, 30]. Among H_2_O_2_-inducible genes, HSPs are related to defense or stress responses [5]. However, the relationship between H_2_O_2_ and HSP20 in grape berry development is not clear. Therefore, a preliminary study on this issue was conducted.

HSP20 proteins as molecular chaperones play an important role in plant growth and development, and deter or reduce the irreversible aggregation of denatured proteins under stress [14, 15]. Although HSP20s block the aggregation and stabilization of non-natural proteins in an ATP-independent manner [17], HSP20s themselves could not refold non-native proteins. Pea Hsp18.1 had to work with the hsp70 system to refold thermally-modified proteins [31]. In recent years, due to the availability of whole genome sequences, HSP20 families have been identified from plants, such as Arabidopsis [11], tomato [32], rice [19], and soybean [22]. However, there are few studies on the HSP20 family in grapes.

Following an integrated approach to detect HSP20s in grape, 48 putative *VvHSP20* genes were identified. These genes were divided into 11 subgroups (CI, CII, CIII, CV, CVI, CVII, MI, MII, ER, CP, and PX/Po). Previous research showed that 12 *HSP20* gene subgroups were identified from Arabidopsis (CI–CVII, MI, MII, ER, CP, and PX/Po) [11, 33]. Likewise, four new nuclear subgroups from rice (CVIII, CIX, CX, and CXI) were reported [9]. However, several subgroups including CIV, CVIII, CIX, CX, and CXI of rice were not identified from the *VvHSP20* genes of grape. One study demonstrated that the CIV subgroup may be involved in coping with diverse stress conditions and may be developmentally regulated [33]. Under normal growth conditions, members of the CVIII subgroup may be heat-induced, while the CX subgroup of genes may be related to specific housekeeping functions [9]. Interestingly, in pepper plants, the HSP20 CIV, CV, CVIII, CIX, CX, and CXI subgroups were found to be absent [18]. In addition, the HSP20 family of rice lacked CIV and CVII subgroups [9]. Therefore, it was easy to see that gene acquisition and loss events are widespread in plant species. The absence of subgroups may be due to the loss of genes during the evolution of *HSP20* genes.

Gene structure plays a crucial role in the evolution of multiple gene families. Our results showed that most of the *VvHSP20* genes (93.8%) had no intron or only one intron of short length. Plants tend to retain genes without introns or with shorter introns [34]. This is consistent with previous reports from pepper [18] and tomato [32], where 97.14 and 83.33% of HSP20 genes, respectively, have no or one short intron. Most *VvHSP20s* in the CII and ER subgroups had no intron, which is consistent with orthologs in pepper, rice, and soybean [18, 19, 22], but the gene structure (exon-intron) of the CI group in grape was different from those in these species, indicating that the intron pattern might not be well preserved among different species. In addition, the stability index of most VvHSP20 proteins was greater than or equal to 40, indicating that most of them were unstable proteins. Instability is believed to be a common feature of stress-responsive proteins, and may also reflect the rapid induction of *VvHSP20* genes [35].

The expression of heat-shock proteins (HSPs) is activated or increased under hight termperature stress, a condition in which HSP20s play important roles in protecting against protein aggregation [14]. HSP20s could be induced not only by environmental stresses, including heat, cold, drought, and salinity, but also by various developmental processes, such as embryogenesis, seed germination, and fruit ripening [22, 36–38]. In this study, the expression of *VvHSP20s* was down-regulated by H_2_O_2_ treatment during fruit development (Fig. 7), in line with our previous research showing hydrogen peroxide can promote the early ripening of ‘Kyoho’ grape [1]. Similarly, *FaHSP17.4* was highly expressed in leaves and flower organs of ‘Fengxiang’ strawberry, but the expression decreased gradually during fruit development [36]. In addition, HSP expression is induced at specific developmental stages in plants. HSP20s were highly expressed in the development stages of zygotic embryonic tissues, and during pollen maturation in rice and tomato [9, 39]. The *NJJS4* gene is a type of HSP20-coding gene, which accumulates in strawberry fruit (*Fragaria* x *ananassa* cv, receptacle) during ripening [40]. Class II sHSP17.4 is expressed at almost all stages of fruit development, and maintained at a high level at the later stage of fruit ripening, while Class II sHSP17.6 reached a peak at the turning stage, and Class I HSP17.7 reached a high level at the pink stage [41]. Four differentially expressed *HSP20* genes were revealed from the RNA-Seq results of tomato fruit (Heize 1706), which were considered to play an important role in fruit development [42]. These observations indicate that HSP20s are associated with fruit development.

ABA plays an important role in promoting fruit ripening. In non-climacteric grape berries, ABA is considered to be the main signal that triggers the onset of maturation-related processes as it peaks at version, accompanied by the beginning of berry softening and skin coloration [43]. ABA content is determined by the dynamic balance of endogenous ABA biosynthesis and catabolism [44]. A previous study showed that 9-*cis*-epoxycarotenoid dioxygenase (NCED) is a key enzyme involved in ABA biosynthesis [45] and CYP707A (an key ABA degradation enzyme) plays a predominant role in ABA catabolism in vivo in strawberry [46, 47]. NCED plays an important role in the ABA-mediated signaling pathway [45, 48]. In order to further understand the relationship between hydrogen peroxide and ABA during grape development, we analyzed the expression of ABA synthesis and degradation-related genes after H_2_O_2_ treatment (Fig. 8). In this study, *NCED3* was found to have low expression at the early stages of fruit development, but to rapidly increase at the K4 stage in the control. However, it reached peak levels at veraison then rapidly decreased at H4 stage. This is consistent with changes in ABA during fruit development, whereby ABA reaches peak levels at the veraison stage and decreases after that [49, 50]. ABA catabolism and biosynthesis are closely linked through feedback and feedforward loops to limit the amount of ABA needed for fruit growth and to rapidly increase the amount of ABA before fruit ripening [47]. The *CYP707A4* gene is highly induced at the H1 stage, then gradully decreases, and finally reaches the lowest values at veraison after H_2_O_2_ treatment. It was previously shown that the expression level of *FveCYP707A4a* was higher in the early stages of fruit development in woodland strawberry [47]. This may be due to a high level of ABA inhibiting early fruit growth [47] and accelerated ABA degradation following hydrogen peroxide treatment.

**Fig. 9:**
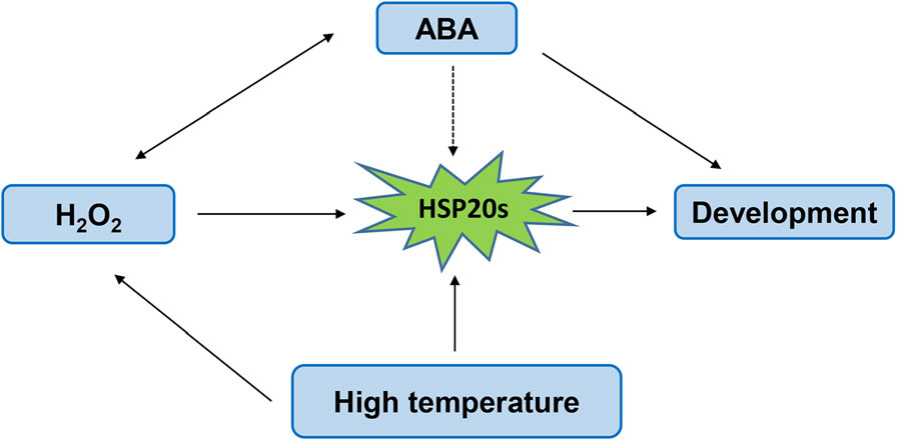
Proposed Model for HSP20 and ABA, H_2_O_2_, high temperature, fruit development

We propose a model for HSP20s and ABA, H_2_O_2_, fruit development, and high temperature (Fig. 9). Under high temperature, HSP20s are activated or increased [51]. In our study, the expression levels of most *HSP20s* were down-regulated during fruit development after H_2_O_2_ treatment (Fig. 7) and H_2_O_2_ treatment promoted early ripening of ‘Kyoho’ grape [1]. In addition, ABA play significant roles in promoting fruit ripening and it is considered that ABA is the main signal triggering the beginning of maturation-related processes. ABA synthesis and metabolism were also affected by H_2_O_2_ (Fig. 8). Interestingly, other studies have shown that ABA induces H_2_O_2_ formation [52]. However, the role of *HSP20s* in this process needs to be further explored.

## Conclusion

In this study, the *HSP20* gene family of grape was comprehensively identified. The phylogenetic relationships, gene structures, conserved motifs, and *cis*-acting elements of 48 *VvHSP20* genes were analyzed, while the expression levels were explored by RNA-Seq and qRT-PCR analysis. A total of 48 HSP20 were divided into 11 subfamilies according to the phylogenetic tree and subcellular localization. The expression levels of *HSP20* genes in grape under H_2_O_2_ treatment were verified by qRT-PCR analysis, providing a basis for further study on the functional analysis of *HSP20* genes during fruit development. Finally, the expression levels of ABA-related genes were verified. We confirmed that H_2_O_2_ indeed affected ABA metabolism and the expression of *HSP20* genes to promote fruit development and ripening.

## Methods

### Identification of *HSP20* genes in grape genome

We downloaded the grapevine reference genome assembly and protein sequences from Ensembl Plants Database (http://plants.ensembl.org/index.html). The grape *HSP20* candidates were identified based on the HMM profile of HSP20 (PF00011). The CDD (https://www.ncbi.nlm.nih.gov/Structure/bwrpsb/bwrpsb.cgi), Pfam and SMART (http://smart.embl-heidelberg.de/) were used to further confirm the conserved HSP20 domain. Finally, 48 HSP20s were identified after removing the redundant sequence without the conserved ACD domain of HSP20 and with the molecular weight outside the range of 15–42 kDa. The Protparam online tools (https://web.expasy.org/protparam/) were used to predict physicochemical properties of HSP20 proteins. The online tool Protcomp (http://linux1.softberry.com/) was used to perform the subcellular localization prediction. The identified *VvHSP20* genes were named according to their positions on pseudomolecules [19].

### Phylogenetic analysis of *HSP20* genes in plants

The amino acid sequences of HSP20s derived from Arabidopsis and tomato and newly identified VvHSP20s were used for phylogenetic analysis. The neighbor joining phylogenetic tree was constructed with the default parameters based on the multiple sequence alignments of the HSP20s amino acid sequences by MEGA 7.0 software.

### Characterization of the amino acid sequences and gene structure of VvHSP20s

The conserved motifs of VvHSP20s were identified using MEME program (version 4.11.2, http://alternate.meme-suite.org/tools/meme), and the parameters were as follows: optimum motif width ranges from 6 to 200 amino acid residues and maximum of 10 misfits. The structures of *VvHSP20* genes in grape was identified using TBtools software [53].

### Chromosomal location and gene duplication of *HSP20* genes

Chromosomal localization information of *VvHSP20* genes was obtained from Ensembl Plants Database (http://plants.ensembl.org/index.html) and the chromosome location images were generated using the MapDraw V2.1 tool (http://mg2c.iask.in/mg2c_v2.0/). The definition of CaHSP20 gene replication is based on the previous research [54]. The duplication events and syntenic analysis of *VvHSP20* genes were determined using MCScanX (Multiple Collinearity Scan) [55] and Circos software, respectively.

### Analysis of *cis*-elements in *VvHSP20* gene promoters

The *cis*-elements were identified from the upstream 2 kb promoter sequences of the *VvHSP20* genes which were submitted to PlantCARE (http://bioinformatics.psb.ugent.be/webtools/plantcare/html/) [56].

### Plant material

Plant samples were collected from the farm of Henan University of Science & Technology, Luoyang, China in 2017. ‘Kyoho’ grape treated with distilled water (containing 0.03% silicon wet-77 surfactant) was naturally grown for 6 years as a control and treated twice with 300 mmol/L H_2_O_2_. The first spraying was conducted at 25 days post anthesis (dpa) in 2017 and the second was 35 dpa. Samples were taken 35 days after flowering and every 10 days until the treated fruits were ripe (Table 2). In addition, the treated berries reached the veraison at 55 dpa. Representative pest-free samples were collected from 5 individual vines of ‘Kyoho’. Thirty samples were randomly selected from each tree to record the phenological data of fruit development.

### RNA extraction and quantitative real-time PCR (qRT-PCR) data analysis

The RNAprep Pure Plant Kit (TIANGEN, Beijing China) was used to isolate total RNA. cDNAs were obtained by total RNA reverse transcription using HiScript® II 1st Strand cDNA Synthesis Kit (Vazyme, Nanjing China). Primers for the *VvHSP20* genes were designed by Primer Premier 5.0 software and listed in Additional file 2: Table S1. The grape *ubiquitin1* gene was used as the reference gene [57, 58] and the expression level of K1 was used as the calibrator. Quantitative real-time PCR was conducted with a total volume of 10 μL of TransStart Top Green qPCR SuperMix kit (TRANSGEN, Beijing China) in CFX96 Real-Time PCR Detection System (Bio-Rad). The relative expression changes of *VvHSP20s* genes were calculated using the 2^-ΔΔCt^ method from three independent replicates [59]. SPSS version 21.0 was employed to analyze the statistical significant differences of the gene expression levels by ANOVA with Duncan’s multiple range test.

The FPKM values of *VvHSP20* genes were from the RNA-Seq data (Accession codes, SRA: PRJNA541089). The average FPKM value of each repetition was converted to log10. Pheatmap (R package) was used to generate the heatmap.

## Supplementary information

**Additional file 1: Figure S1.** Syntenic relationships among *VvHSP20s* genes. Different colors represent different chromosomes. Lines of different colors represent different tandem duplication genes.

**Additional file 2: Table S1.** Primers used for the qRT-PCR reactions.

## Data Availability

In this study, the grape genome sequence used to identify *HSP20* genes were downloaded from Ensembl Plants Database (http://plants.ensembl.org/index.html). Expression data of *VvHSP20* genes in grape used in this study can be accessed via the NCBI SRA database with accession numbers of PRJNA541089 from 5th May 2020 onwards, as until then there is an embargo due to a complementary manuscript. Until then, these sequences are available from the corresponding author upon reasonable request.

## Abbreviations

ABA: Amino acid
ACD: Alpha-crystallin domain
CRI: Conserved region I
CRII: Conserved region II
HSP20s: Small heat shock proteins
PSII: Photosystem II
