# Supplementary figures and images for: Genome-wide identification of small heat-shock protein (HSP20) gene family in grape and expression profile during berry development
Source: BMC Plant Biol. 2019 Oct 17;19:433. doi: 10.1186/s12870-019-2031-4 (PMC6798335; doi:10.1186/s12870-019-2031-4)

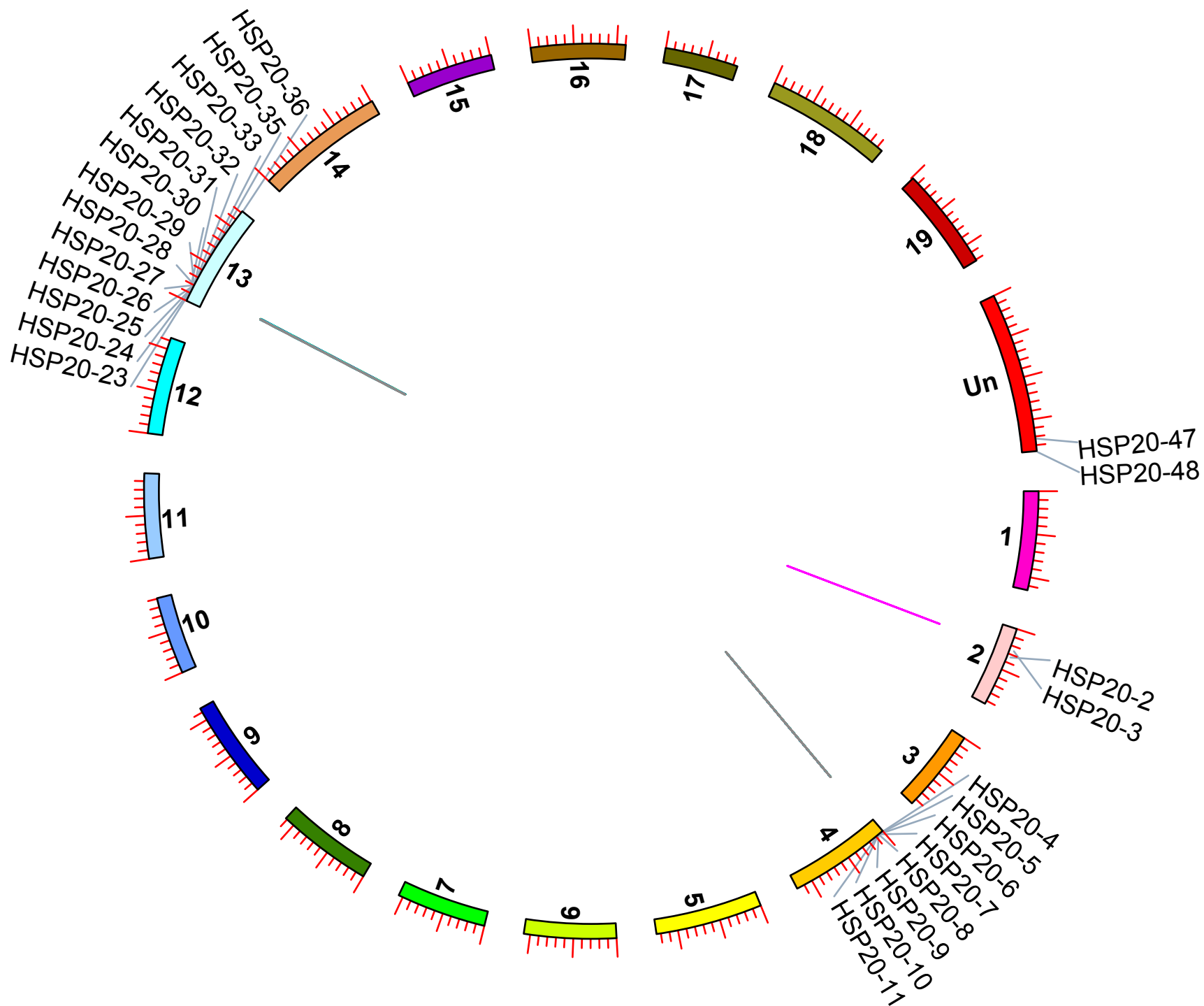

Supplement: Supplementary file 1 — Additional file 1: Figure S1. Syntenic relationships among VvHSP20s genes. Different colors represent different chromosomes. Lines of different colors represent different tandem duplication genes. [file 12870_2019_2031_MOESM1_ESM.pdf]
